# First-in-Human Use of Intravascular Lithotripsy to Facilitate Mitral Balloon Commissurotomy After Prior Annuloplasty Band Repair

**DOI:** 10.1016/j.jaccas.2025.106562

**Published:** 2026-02-18

**Authors:** Tooba Salar, David C. Corteville, Elizabeth Carol Lee, Jeremiah Depta

**Affiliations:** a Rochester General Hospital, Rochester, New York, USA; b Medical College of Wisconsin, Milwaukee, Wisconsin, USA

**Keywords:** annuloplasty band, intravascular lithotripsy, mitral stenosis, mitral valve surgery, percutaneous mitral balloon commissurotomy

## Abstract

**Background:**

Patients with severe mitral stenosis (MS) after surgical repair often face limited therapeutic options owing to high surgical risk and complex valve anatomy.

**Case Summary:**

A 79-year-old woman with prior surgical mitral valve repair presented with severe MS. Given her high surgical risk and extensive valvular calcification, she was not a candidate for redo surgical mitral valve, percutaneous mitral balloon commissurotomy (PMBC), or transcatheter mitral valve-in-ring. Intravascular lithotripsy (IVL)–facilitated PMBC was performed, with postprocedural improvement in gradients and symptoms.

**Discussion:**

IVL was used to modify calcific leaflets, enabling successful commissurotomy in complex postsurgical anatomy. Comparative hemodynamic data are needed to clarify IVL's contribution.

**Take-Home Messages:**

IVL can be used off-label for severe MS with prior incomplete annuloplasty band where surgery, transcatheter mitral valve-in-ring, and PMBC are not viable options. IVL-facilitated PMBC still remains investigational; larger studies and longer term follow-ups are required to define safety, durability, and patient outcomes.

## Visual Summary

**Figure:**
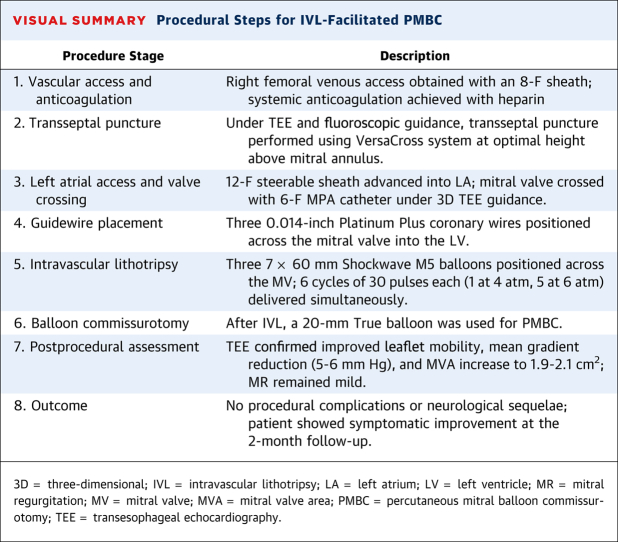

Severe mitral stenosis (MS) presents a significant clinical challenge, particularly in an aging population with complex comorbidities and anatomical considerations. Surgical mitral valve replacement, though definitive, often carries prohibitive risks in elderly, frail patients with high surgical risk scores. Although percutaneous mitral balloon commissurotomy (PMBC) is a guideline-endorsed option for select patients with pliable rheumatic valves, its role in postsurgical or calcific MS is limited. Transcatheter mitral valve-in-ring (TMViR) therapy may be anatomically unsuitable, especially in the setting of incomplete or flexible annuloplasty rings. Intravascular lithotripsy (IVL), a novel technique that applies acoustic shockwaves to modify calcium, is emerging as a potential adjunctive therapy to facilitate PMBC in heavily calcified mitral valves. Early case series have demonstrated its feasibility in native MS. We describe a case of IVL-facilitated PMBC in a patient with severe symptomatic MS and a prior annuloplasty band. This case illustrates the procedural strategy and short-term outcome of a novel percutaneous approach in a patient deemed unsuitable for both surgical and conventional transcatheter options.

**Take-Home Messages**

• IVL can be used off-label for severe mitral stenosis with prior incomplete annuloplasty band where surgery, transcatheter mitral valve-in-ring, and PMBC are not viable options.
• IVL-facilitated PMBC still remains investigational; larger studies and longer term follow-ups are required to define safety, durability, and patient outcomes.

## Case Presentation

A 79-year-old woman presented with NYHA functional class III symptoms. She had a history of breast cancer treated with chemotherapy and underwent surgical mitral valve repair in 2008 using a 29-mm Duran flexible annuloplasty band.

**Figure 1:**
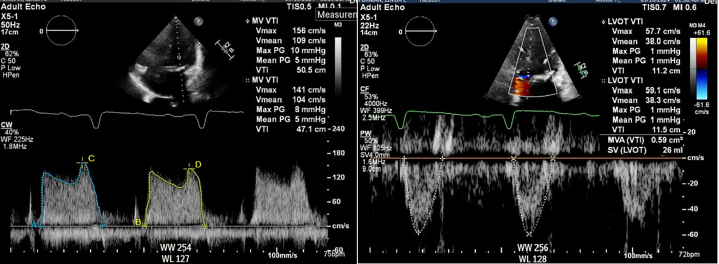
Transthoracic Echocardiography Showing Mitral Valve Area of 0.59 cm^2^ by Velocity Time Integral

**Figure 2:**
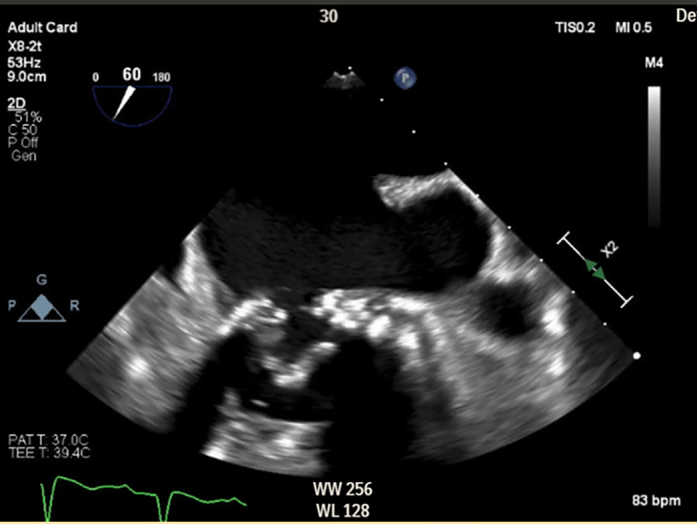
Transesophageal Echocardiography Showing Severe Mitral Calcification and Leaflet Immobility

**Figure 3:**
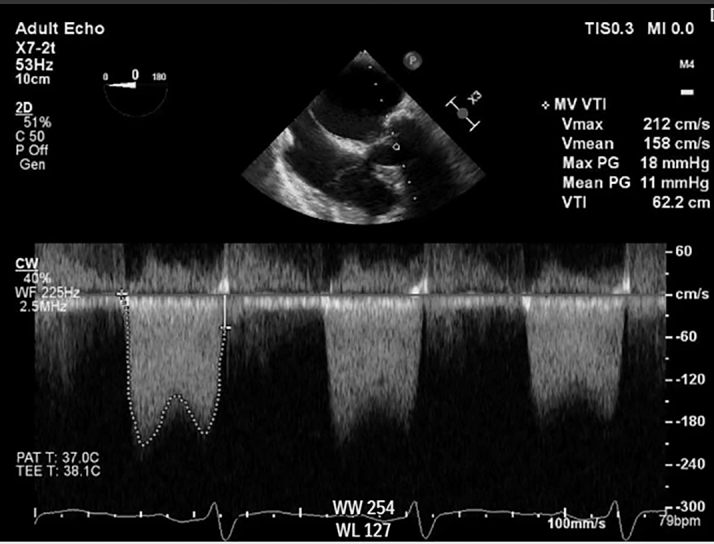
Transesophageal Echocardiography Showing Mean Mitral Valve Gradient 11 mm Hg at 79 beats/min

**Figure 4:**
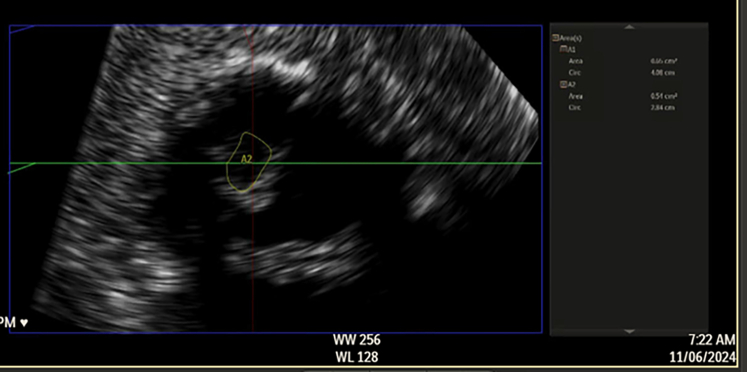
Transesophageal Echocardiography Showing Mitral Valve Area 0.51 cm^2^ by Planimetry

**Figure 5:**
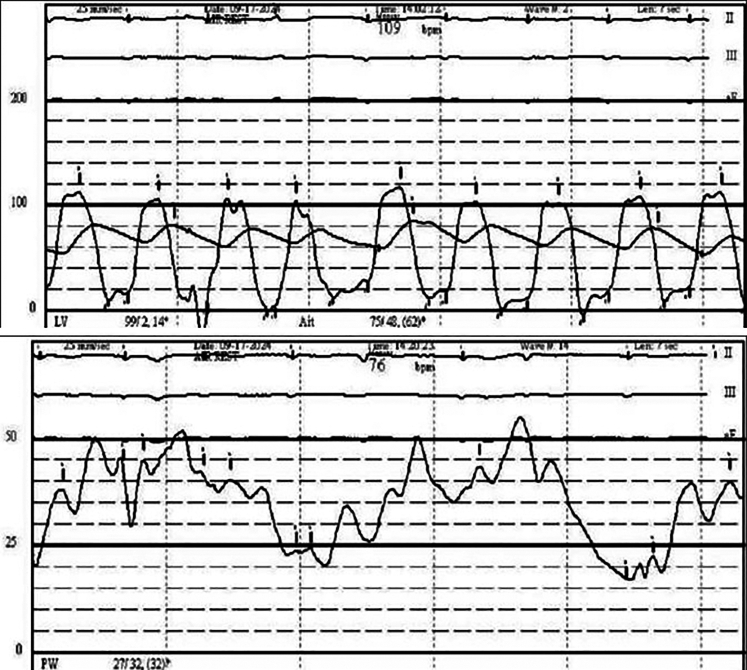
Right Heart Catheterization With Pulmonary Capillary Wedge Pressure 32 mm Hg and Transmitral Gradient of ∼18 mm Hg

**Figure 6:**
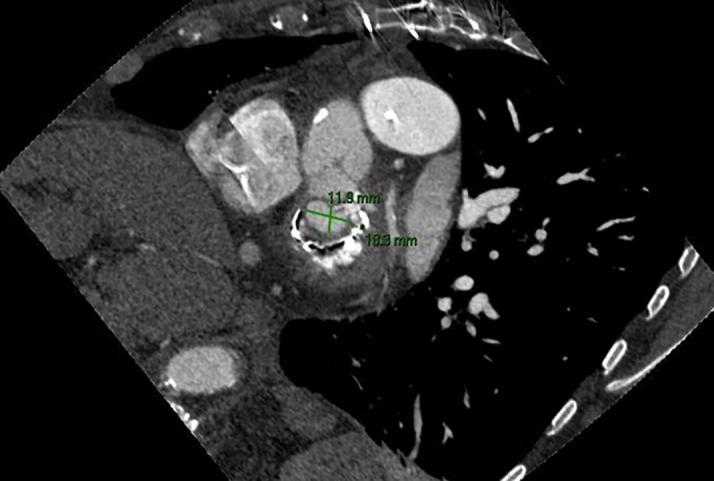
Gated Cardiac Computed Tomography With Mitral Annular Dimensions 2 × 1.8 cm

Transthoracic echocardiography demonstrated severe MS with thickening and calcification of both leaflets. The mitral valve area was 0.52 cm^2^ by velocity time integral (Figure 1). Transesophageal echocardiography (TEE) confirmed markedly restricted leaflet mobility (Figure 2, Video 1), mean diastolic gradient across the mitral valve was 11 mm Hg at 79 beats/min (Figure 3), and the mitral valve area was calculated between 0.5-0.6 cm^2^ by three-dimensional (3D) planimetry (Figure 4). Right heart catheterization revealed a right atrial pressure of 13 mm Hg, right ventricular pressure of 55/10 mm Hg (mean: 41 mm Hg), and pulmonary capillary wedge pressure of 32 mm Hg. Left ventricular end-diastolic pressure was 14 mm Hg, and mean transmitral gradient was 18 mm Hg (Figure 5). Left heart catheterization demonstrated normal coronary arteries. Gated cardiac computed tomography showed severe mitral annular calcification, with annular dimensions measuring 2 × 1.8 cm and absence of calcium anteriorly (Figure 6).

Given the patient's advanced age, frailty, and elevated Society of Thoracic Surgeons predicted risk of mortality score (8.83%), she was deemed high risk for redo surgical mitral valve replacement. PMBC was considered; however, the presence of extensive valvular calcification and a high Wilkins score (>8) rendered the procedure high risk. TMViR therapy was also contemplated, but the incomplete and flexible nature of the prior annuloplasty band precluded this option owing to risk of inadequate anchoring and prosthesis instability.

After multidisciplinary heart team discussion, the decision was made to pursue off-label PMBC facilitated by IVL to modify the valvular calcium and improve leaflet mobility.

## Procedural Details

Access was obtained via an 8-F sheath placed in the right femoral vein. Cerebral embolic protection was not performed owing to tortuous anatomy. Systemic anticoagulation was achieved using intravenous heparin to maintain an activated clotting time >250 seconds.

A 0.035-inch VersaCross wire (Boston Scientific) was advanced into the superior vena cava. An 8.5-F VersaCross transseptal sheath and dilator were then positioned in the superior vena cava. Under TEE guidance, the system was advanced to tent the interatrial septum at an appropriate height above the mitral annulus. After confirmation of optimal positioning on multiple TEE views, radiofrequency energy was applied to cross the septum. The dilator was advanced across the interatrial septum, and the wire was further advanced into the left atrium. The dilator was subsequently removed, and a 12-F steerable catheter was introduced into the left atrium over the wire. The VersaCross wire was then removed.

Using a straight 6-F multipurpose A (MPA) guide catheter, the mitral valve was crossed with the aid of 3D TEE, and the steerable catheter was advanced into the left ventricle. Three 0.014-inch Platinum Plus coronary wires (Boston Scientific) were positioned across the mitral valve into the left ventricle.

**Figure 7:**
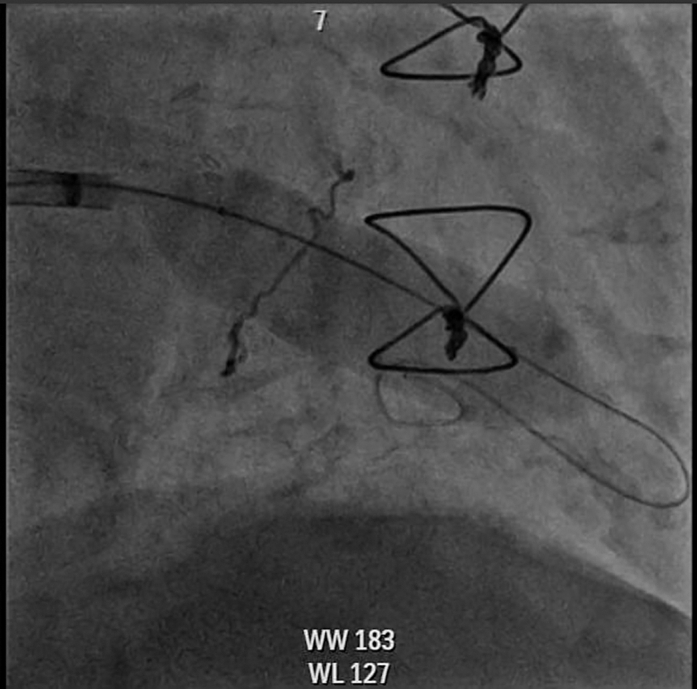
Cineangiography of Percutaneous Mitral Balloon Commissurotomy With 20-mm True Balloon

IVL was performed using three 7.0 × 60 mm Shockwave M5 balloons (Shockwave Medical). Each balloon delivered 6 cycles of treatment (30 pulses per cycle), with the first cycle at 4 atm and the remaining 5 cycles at 6 atm, for a total of 180 pulses per balloon (Video 2). After lithotripsy, the balloons were removed. The steerable sheath was advanced into the left ventricle, and the coronary wires were withdrawn. A pigtail catheter was then advanced into the left ventricle and exchanged for a Safari wire (Boston Scientific). Balloon commissurotomy was subsequently performed using a 20-mm True balloon (Becton Dickinson) (Figure 7, Video 3).

**Figure 8:**
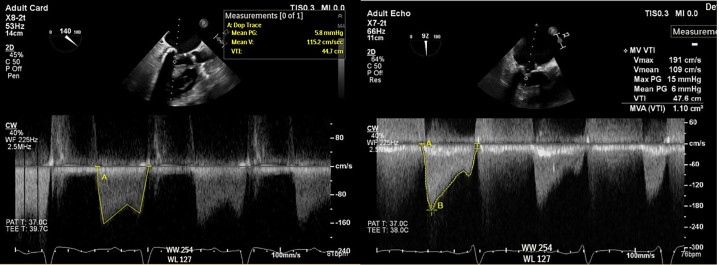
Postprocedural MV Gradients on Transesophageal Echocardiography (A) MV gradient after IVL with improvement to 5.8 mm Hg. (B) Similar gradients after PMBC. IVL = intravascular lithotripsy; MV = mitral valve; PMBC = percutaneous mitral balloon commissurotomy.

**Figure 9:**
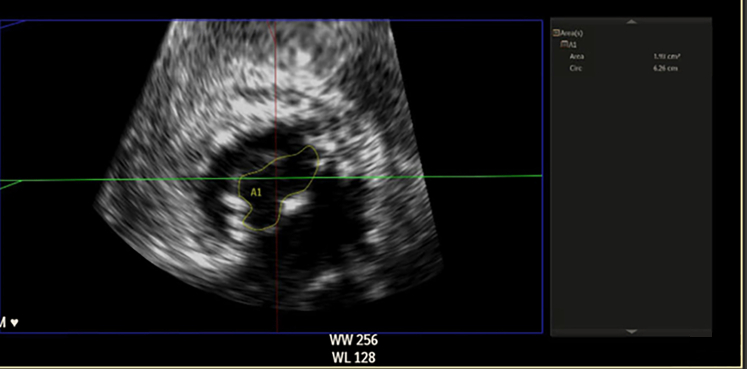
Improvement in 3-Dimensional Mitral Valve Area to 1.9 cm^2^

**Figure 10:**
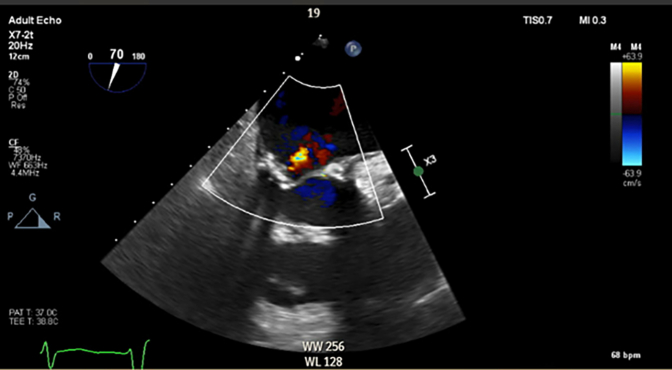
Mild Mitral Regurgitation

**Figure 11:**
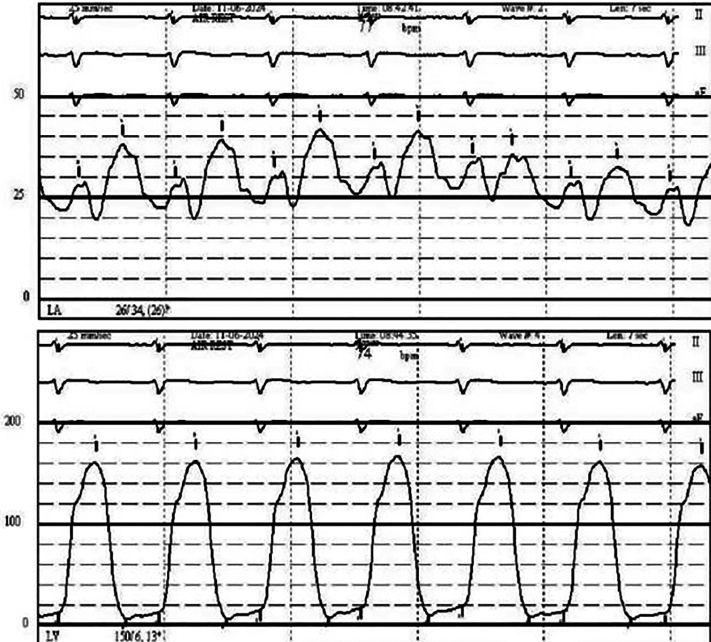
Postprocedural Pulmonary Capillary Wedge Pressure Improved to 25 mm Hg With Estimated Transmitral Gradient ∼12 mm Hg

Postprocedural TEE demonstrated improved mobility of the mitral valve leaflets with a reduction in the mean transmitral gradient to 5-6 mm Hg (Figure 8) and an increase in mitral valve area to 1.9-2.1 cm^2^ by 3D planimetry (Figure 9, Video 4). Mitral regurgitation remained mild (Figure 10). Hemodynamic improvement was evidenced by a reduction in mean left atrial pressure to 17 mm Hg postprocedure (Figure 11).

No immediate postprocedural complications were noted, and the patient recovered without any neurological sequalae. She was seen at the 2-month follow-up with improvement in symptoms.

## Discussion

This case presented unique technical and clinical challenges given the patient's anatomy, prior surgical mitral repair, and high surgical risk. The patient's Society of Thoracic Surgeons score of 8.83%, combined with frailty and the risks associated with redo sternotomy, rendered her a poor candidate for surgical mitral valve replacement.

Although PMBC is a recognized treatment option for select patients with severe MS, its utility is limited in the setting of heavily calcified, nonpliable valves, especially when the Wilkins score is >8. In such cases, PMBC carries an increased risk of leaflet tearing and resultant severe mitral regurgitation, which made it a less favorable standalone option here.

TMViR using the balloon-expandable Edwards Sapien 3 valve is commercially available for patients with failed surgical repairs involving complete annuloplasty rings.^1^ However, our patient had an incomplete and flexible 29-mm Duran band with no significant anterior mitral annular calcification (MAC) (Figure 6), eliminating the possibility of secure valve anchoring and disqualifying her from TMViR or valve-in-MAC approaches.

In light of these limitations, an off-label approach using IVL-facilitated PMBC was pursued. IVL is a novel technique that delivers acoustic shockwaves via balloon catheters to fracture valvular calcium, enhancing leaflet mobility and enabling safer balloon commissurotomy.^2^ Although initially described for MAC-related MS^3^ and rheumatic MS,^4^ this case demonstrates its feasibility in a more complex postannuloplasty setting.

We used three 7 × 60 mm Shockwave M5 IVL balloons simultaneously to deliver focused energy across the mitral valve before balloon dilation. This approach is used to increase pliability of the valve and reduce risk of acute MR, and it has also been seen in prior case series, including the one by Giustino et al,^5^ who reported favorable hemodynamic outcomes with 7- to 12-mm–diameter balloons used in parallel.

Notably, Sharma et al^4^ uniquely assessed invasive hemodynamic parameters immediately after IVL and before PMBC and observed significant reductions in transmitral gradients, suggesting a direct mechanical benefit of IVL on valve compliance. Although we did not assess the invasive hemodynamic effect of IVL alone, gradients on TEE improved right after IVL (Figure 8A) and remained almost similar after PMBC (Figure 8B). Mitral valve area increased, indicating procedural success (Videos 1 and 4), with only mild mitral regurgitation. Residual mitral valve mean gradient was ∼6 mm Hg, consistent with prior case series reporting 33% of patients with a residual gradient >5 mm Hg.^5^ Postprocedural hemodynamic assessment also revealed improvement in transmitral gradient.

No immediate complications such as pericardial effusion or complete heart block were noted. Mitral regurgitation was only mild. Cerebral embolic protection was not used given anatomic constraints; however, the patient experienced no neurologic complications. The occurrence of stroke in prior reports where embolic protection was omitted underscores the importance of using cerebral protection when feasible.^5^

Despite its feasibility, IVL-guided PMBC is still an investigational technique and should be regarded as such. This case adds to the emerging experience supporting IVL-facilitated PMBC as an option for patients with complex mitral valve pathology, including those with prior surgical repair. However, the current evidence is limited to isolated case reports and small case series. Larger prospective studies are needed to validate the safety, efficacy, and long-term outcomes of this off-label technique in diverse clinical scenarios before broader clinical adoption.

As we did not evaluate the hemodynamic effect of IVL alone (before PMBC), we cannot conclusively determine the incremental contribution of IVL on gradient reduction alone. Longer term follow-up data and comparative studies (IVL alone vs IVL plus PMBC) are required to further establish the role of this strategy as well as validate safety.

## Conclusions

IVL-facilitated PMBC may serve as a feasible and safe off-label strategy for patients with severe MS and prior incomplete annuloplasty who are unsuitable for surgical or conventional transcatheter therapies. Although this approach resulted in significant gradient reduction and symptomatic improvement, larger studies are warranted to validate safety, durability, and incremental benefit of IVL through longer term studies.

## Funding Support and Author Disclosures

Dr Depta has provided consultation for Edwards Lifesciences and Boston Scientific. All other authors have reported that they have no relationships relevant to the contents of this paper to disclose.

## Figures and Tables

**Visual Summary:** Procedural Steps for IVL-Facilitated PMBC

Procedure Stage	Description
1. Vascular access and anticoagulation	Right femoral venous access obtained with an 8-F sheath; systemic anticoagulation achieved with heparin
2. Transseptal puncture	Under TEE and fluoroscopic guidance, transseptal puncture performed using VersaCross system at optimal height above mitral annulus.
3. Left atrial access and valve crossing	12-F steerable sheath advanced into LA; mitral valve crossed with 6-F MPA catheter under 3D TEE guidance.
4. Guidewire placement	Three 0.014-inch Platinum Plus coronary wires positioned across the mitral valve into the LV.
5. Intravascular lithotripsy	Three 7 × 60 mm Shockwave M5 balloons positioned across the MV; 6 cycles of 30 pulses each (1 at 4 atm, 5 at 6 atm) delivered simultaneously.
6. Balloon commissurotomy	After IVL, a 20-mm True balloon was used for PMBC.
7. Postprocedural assessment	TEE confirmed improved leaflet mobility, mean gradient reduction (5-6 mm Hg), and MVA increase to 1.9-2.1 cm^2^; MR remained mild.
8. Outcome	No procedural complications or neurological sequelae; patient showed symptomatic improvement at the 2-month follow-up.

3D = 3-dimensional; IVL = intravascular lithotripsy; LA = left atrium; LV = left ventricle; MR = mitral regurgitation; MV = mitral valve; MVA = mitral valve area; PMBC = percutaneous mitral balloon commissurotomy; TEE = transesophageal echocardiography.

## Appendix

Video 1 Three-Dimensional En Face View of the Mitral Valve From the Left Atrium Showing Mitral Stenosis With Calcification of the Commissures and Leaflets

Video 2 Three-Dimensional En Face View of 3 IVL Balloons Being Deployed Simultaneously in the Mitral Valve

Video 3 Mitral Balloon Commissurotomy Balloon Being Deployed

Video 4 Postprocedural Three-Dimensional Imaging
